# Association of PSQI and SCL-90 scores with early clinical identification of posttraumatic stress disorder after emergency trauma surgery

**DOI:** 10.3389/fpsyg.2026.1730054

**Published:** 2026-05-18

**Authors:** Zhang Bu, Yuqian Zhou, Feng Xu, Shan Xu

**Affiliations:** 1 Department of Emergency Medicine, The First Affiliated Hospital of Soochow University, Suzhou, Jiangsu, China; 2 Soochow University Campus Hospital, Soochow University, Suzhou, Jiangsu, China

**Keywords:** emergency trauma surgery, injury severity score, Pittsburgh sleep quality index, post-traumatic stress disorder, post-traumatic stress disorder checklist-civilian version, symptom checklist-90

## Abstract

**Objective:**

To evaluate the association of Pittsburgh Sleep Quality Index (PSQI) and Symptom Checklist-90 (SCL-90) scores with post-traumatic stress disorder (PTSD) identified 1 month after emergency trauma surgery.

**Methods:**

In this retrospective convenience sample, 98 patients with PTSD identified 1 month after emergency trauma surgery (March 2022 to March 2024) were assigned to the PTSD group, and 110 patients without PTSD were included as the non-PTSD group. Baseline characteristics, Injury Severity Score (ISS), and Post-traumatic Stress Disorder Checklist-Civilian Version (PCL-C) were recorded. PSQI and SCL-90 scores were assessed at the same postoperative time point. ROC analysis was used to evaluate the discriminatory performance and optimal cut-off values of these scores for early clinical identification of PTSD. Correlation analyses examined relationships among PSQI, SCL-90, PCL-C, and ISS. Multivariate logistic regression was used to identify factors independently associated with PTSD. A *post hoc* power analysis was additionally performed for baseline variables with *P* > 0.05, and a 1:1 propensity score matching sensitivity analysis was conducted.

**Results:**

The PTSD group had significantly lower proportions of married individuals, those with a bachelor's degree or higher, and those with monthly income >5000 RMB, along with higher ISS and PCL-C scores (all P < 0.05). PSQI and SCL-90 scores were significantly higher in the PTSD group (*P* < 0.001). The combined PSQI and SCL-90 model showed the largest area under the curve for PTSD identification (AUC = 0.937, 95%CI: 0.895–0.966), with an optimal probability cut-off of 0.462, 89.80% sensitivity, and 84.55% specificity. The optimal cut-off values for PSQI and SCL-90 were 11.5 and 128.5, respectively. PSQI and SCL-90 scores were positively correlated with ISS (*r* = 0.742 and *r* = 0.586) and PCL-C scores (*r* = 0.625 and *r* = 0.493; all *P* < 0.001). Multivariate analysis confirmed that education, monthly income, PSQI score, and SCL-90 score were independently associated with PTSD status (all P < 0.05). The matched sensitivity analysis yielded consistent results.

**Conclusion:**

PSQI and SCL-90 scores are closely associated with PTSD identified 1 month after emergency trauma surgery. Their combined assessment showed high value for early identification and risk stratification and may support postoperative psychological screening in trauma populations.

## Introduction

Post-traumatic stress disorder (PTSD) is a trauma-related mental disorder that may occur after exposure to actual or threatened death, severe injury, or other catastrophic experiences (Maercker et al., 2022; Thakur et al., 2022). Traffic accidents, fires, explosions, and other emergency injuries remain important causes of both physical trauma and subsequent psychological morbidity (Yimer et al., 2023). In trauma populations, PTSD can substantially impair rehabilitation, social functioning, and quality of life, making timely postoperative identification clinically important.

Sleep is a core biological process that maintains circadian homeostasis and neurobehavioral adaptation. Increasing evidence indicates that sleep disruption, neuroendocrine imbalance, immune dysregulation, and autonomic dysfunction are closely involved in PTSD-related pathophysiology (Agorastos and Olff, 2021; Delic et al., 2021). Sleep-related symptoms are among the most prominent complaints in PTSD, and the Pittsburgh Sleep Quality Index (PSQI) is one of the most widely used clinical instruments for quantifying subjective sleep quality (Buysse et al., 1989).

Broad psychological distress is another important dimension after trauma. The Symptom Checklist-90 (SCL-90) is a commonly used multidimensional screening instrument for somatization, anxiety, depression, interpersonal sensitivity, and related symptoms (Kostaras et al., 2020). Although PSQI and SCL-90 have each been used in psychiatric assessment, evidence regarding their combined value for identifying PTSD after emergency trauma surgery remains limited. Because trauma recovery often reflects the joint influence of sleep disturbance, affective dysregulation, and stress-system activation, combined assessment may better capture the multidimensional psychological burden seen in the early postoperative period. On this basis, the present study examined the association of PSQI and SCL-90 scores with PTSD identified 1 month after emergency trauma surgery.

## Materials and methods

### Study population

Retrospectively, 113 patients who were identified as having PTSD 1 month after emergency trauma surgery in our hospital from March 2022 to March 2024 were screened, and 98 patients were finally included in the PTSD group according to the inclusion and exclusion criteria. Another 132 patients without PTSD after admission for emergency trauma surgery during the same period were screened, and 110 were finally included in the non-PTSD group. This was a retrospective convenience sample derived from consecutive eligible cases during the study period. The study followed the ethical guidelines of the Declaration of Helsinki and complied with relevant EQUATOR recommendations. The study was approved by the Ethics Committee of our hospital.

### Inclusion and exclusion criteria

Inclusion criteria: (1) all patients in the PTSD group met the diagnostic criteria of PTSD; (2) >20 years old; (3) no other major stressful events occurred within 1 year; (4) normal intelligence; (5) normal consciousness, and can communicate with healthcare personnel in a stable and smooth manner;

Exclusion criteria: (1) family history of mental disorders; (2) history of psychiatric diseases; (3) patients who cannot independently conduct questionnaire assessment; (4) loss of consciousness; (5) patients who have entered the exacerbation or acute phase of other diseases; (6) patients with organic brain damage; (7) previous underlying diseases.

### PTSD diagnosis

Reference was made to the Diagnostic and Statistical Manual of Mental Disorders, Fifth Edition (DSM-5), as the diagnostic standard for PTSD (American Psychiatric Association, 2013). The diagnostic framework included exposure to a traumatic event; intrusive symptoms such as involuntary recollections or trauma-related nightmares; persistent avoidance; negative alterations in cognition and mood; hyperarousal symptoms such as irritability, exaggerated startle, difficulty concentrating, and sleep disturbance; and a duration of at least 1 month accompanied by clinically significant functional impairment.

### Sample and data collection

Baseline information on age, gender, body mass index (BMI), history of smoking, history of alcohol consumption, family history of alcohol dependence, marital status, education, monthly income, healthcare payment method, occupation, traumatic experiences, religious affiliation, ISS, and PCL-C was collected from medical records. PSQI and SCL-90 were assessed at 1 month after emergency trauma surgery, which was the same time point used for PTSD status ascertainment.

### Injury severity score (ISS)

The ISS is a standardized anatomical trauma severity index based on six body regions and the Abbreviated Injury Scale, with a total score ranging from 1 to 75 (Dehouche, 2022). An ISS >16 generally indicates major trauma and is associated with a markedly increased risk of adverse outcomes. In the present study, ISS was used to characterize injury burden at admission.

### Post-traumatic stress disorder check-list-civilian version (PCL-C)

All subjects were assessed 1 month after emergency trauma surgery using the PCL-C, a 17-item scale covering re-experiencing, avoidance/numbing, and hyperarousal symptoms (Weathers et al., 1993). Each item is scored from 1 to 5, yielding a total score of 17–85, with higher scores indicating greater PTSD symptom severity.

### Pittsburgh sleep quality index (PSQI)

All subjects were assessed by the PSQI 1 month after emergency trauma surgery. The PSQI was developed by Buysse et al. (1989) and includes seven components: subjective sleep quality, sleep latency, sleep duration, habitual sleep efficiency, sleep disturbance, use of sleep medication, and daytime dysfunction (Buysse et al., 1989). The total score ranges from 0 to 21, with higher scores indicating poorer sleep quality.

### Symptom checklist 90 (SCL-90) score

All subjects were assessed by the SCL-90 1 month after emergency trauma surgery. The SCL-90 includes nine major symptom dimensions: somatization, obsessive-compulsive symptoms, interpersonal sensitivity, depression, anxiety, hostility, phobic anxiety, paranoid ideation, and psychoticism (Kostaras et al., 2020). A total of 90 items are scored from 1 to 5, and higher total scores indicate more severe overall psychological distress.

### Statistical analysis

Data were statistically analyzed and plotted using SPSS 21.0, MedCalc 22.2, and GraphPad Prism 9.0. Continuous variables conforming to a normal distribution are presented as mean ± standard deviation and were compared using the independent-samples *t* test; non-normally distributed variables are presented as median (interquartile range) and were compared using the Mann-Whitney U test. Categorical variables are presented as n (%) and were compared using the chi-square test. ROC curves were used to evaluate the discriminatory performance of PSQI, SCL-90, and their combination, and optimal cut-off values were determined by the Youden index. The combined indicator was generated from a binary logistic regression model using PSQI and SCL-90 as covariates. Correlations were examined using Pearson or Spearman analysis as appropriate. Variables with *P* < 0.05 in the univariate analysis were entered into multivariate logistic regression to identify factors independently associated with PTSD status. Because this was a retrospective convenience sample, a *post hoc* power analysis was additionally performed for baseline variables with *P* > 0.05. Observed effect sizes for age, sex, BMI, smoking history, drinking history, family history of alcohol dependence, medical payment method, occupation, trauma experience, and religious affiliation corresponded to estimated powers ranging from 0.11 to 0.43, indicating that these null baseline findings should be interpreted cautiously. To reduce confounding bias, a 1:1 nearest-neighbor propensity score matching sensitivity analysis with a caliper of 0.20 was performed using age, sex, BMI, marital status, educational attainment, monthly income, and ISS. *P* < 0.05 was considered statistically significant.

## Results

### Characteristics of baseline data of the enrolled population

Baseline data on age, gender, BMI, smoking history, drinking history, family history of alcohol dependence, marital status, education level, monthly income, medical payment method, occupation, traumatic experiences, religious affiliation, ISS, and PCL-C were compared between groups. The PTSD group had lower proportions of married individuals, participants with an undergraduate education or above, and participants with monthly income >5000 RMB, while ISS and PCL-C scores were significantly higher than those in the non-PTSD group (all *P* < 0.05). Other baseline variables were not significantly different between groups; however, *post hoc* power for these negative comparisons was limited (Table 1).

**Table 1:** General information of the enrolled population.

Variable	PTSD group (*N* = 98)	Non-PTSD group (*N* = 110)	Z/t/χ^2^	*P*
Age (years)	28 (26, 31)	29 (26, 31)	0.048	0.962
Sex (m/f)	39/59	56/54	2.580	0.108
BMI (kg/m^2^)	23.441 ± 0.60	23.321 ± 0.51	0.571	0.569
Smoking history (*N*, %)			2.844	0.092
Yes	21 (21.43%)	35 (31.82%)		
No	77 (78.57%)	75 (68.18%)		
Drinking history (*N*, %)			0.878	0.349
Yes	16 (16.33%)	13 (11.82%)		
No	82 (83.67%)	97 (88.18%)		
Family history of alcohol dependence (*N*, %)			0.214	0.644
Yes	4 (4.08%)	6 (5.45%)		
No	94 (95.92%)	104 (94.55%)		
Marital status (*N*, %)			6.910	0.032
Single or unmarried	23 (23.47%)	20 (18.18%)		
**Married**	34 (34.69%)	58 (52.73%)		
Divorced or widowed	41 (41.84%)	32 (29.09%)		
Educational level (*N*, %)			8.155	0.004
High school and below	55 (56.12%)	40 (36.36%)		
Undergraduate and above	43 (43.88%)	70 (63.64%)		
Monthly income (*N*, %)			8.662	0.003
*≤5000 RMB*	48 (48.98%)	32 (29.09%)		
*>5000 RMB*	50 (51.02%)	78 (70.91%)		
Medical payment method (*N*, %)			2.092	0.148
*Medical insurance*	74 (75.51%)	73 (66.36%)		
*Self-funded*	24 (24.49%)	37 (33.64%)		
Occupation (*N*, %)			2.220	0.136
High-stress occupations	40 (40.82%)	34 (30.91%)		
Non-high-stress occupations	58 (59.18%)	76 (69.09%)		
Trauma experience (*N*, %)			0.859	0.651
*Traffic accident*	53 (54.08%)	61 (55.45%)		
*Blast*	21 (21.43%)	18 (16.36%)		
*Natural disaster*	24 (24.49%)	31 (28.18%)		
Religious affiliation (*N*, %)			1.370	0.242
Yes	14 (14.29%)	10 (9.09%)		
No	84 (85.71%)	100 (90.91%)		
ISS	11 (10, 12)	7 (7, 8)	12.018	<0.001
PCL-C score	43.566 ± 0.76	30.616 ± 0.11	14.433	<0.001

BMI, body mass index; ISS, injury severity score; PCL-C, post-traumatic stress disorder checklist-civilian version. Kolmogorov-Smirnov testing was used for normality assessment. Normally distributed variables are expressed as mean ± standard deviation and were compared using the independent-samples *t* test; non-normally distributed variables are expressed as median (interquartile range) and were compared using the Mann-Whitney U test; categorical variables are expressed as *n* (%) and were compared using the chi-square test.

### Elevated PSQI and SCL-90 scores in PTSD patients after emergency trauma surgery

**Figure 1:**
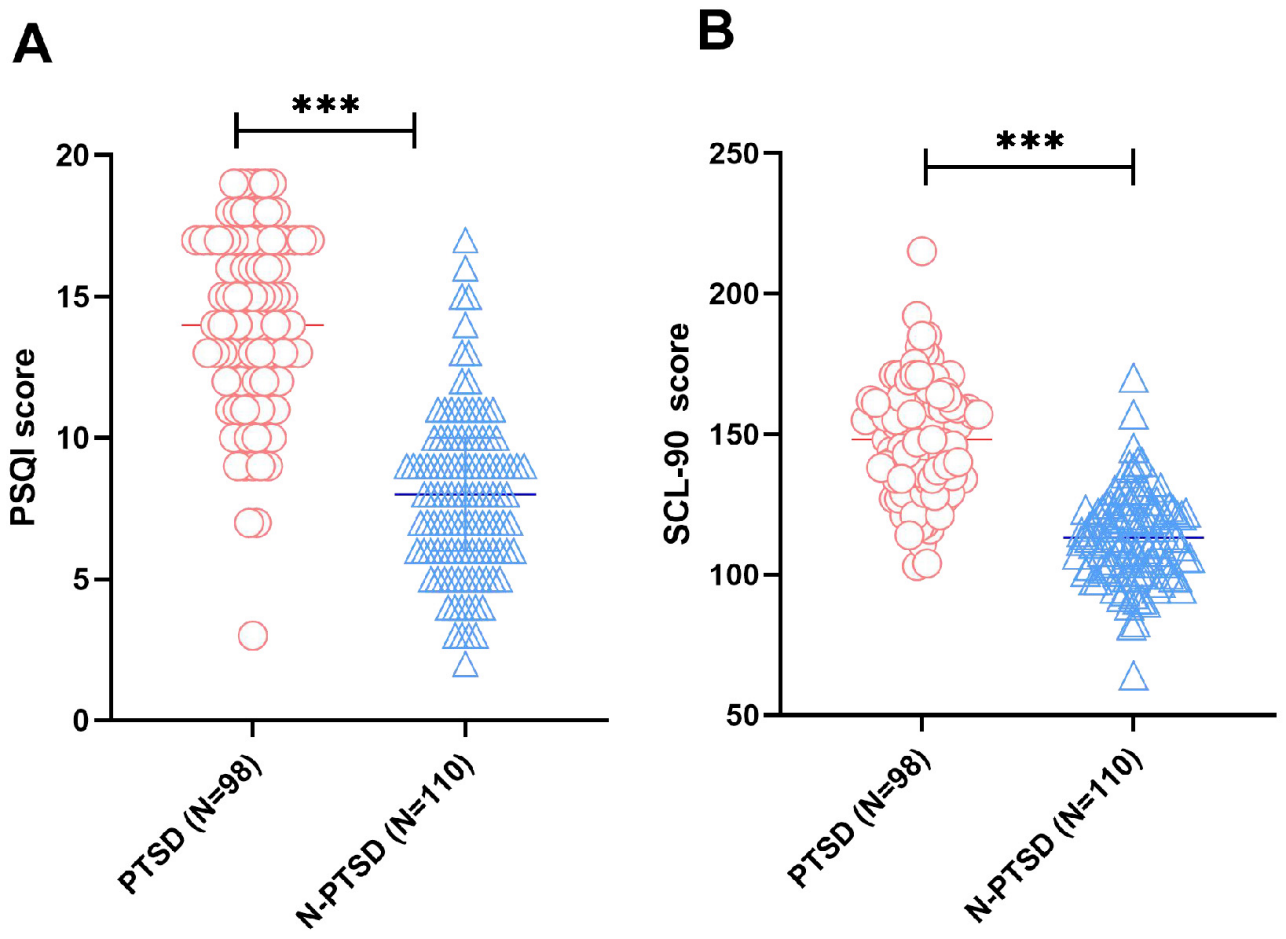
*Elevated PSQI and SCL-90 scores in patients with PTSD after emergency trauma surgery*. **(A)** PSQI scores; **(B)** SCL-90 scores. Group comparisons were performed after assessment of distributional characteristics. Normally distributed measurements are expressed as mean ± standard deviation and were compared using the independent-samples *t* test. Non-normally distributed measurements are expressed as median (interquartile range) and were compared using the Mann-Whitney U test. ***P < 0.001.

PSQI and SCL-90 scores were further compared between groups 1 month after emergency trauma surgery. As shown in Figure 1, the PSQI scores of the PTSD group and non-PTSD group were 14 (12, 17) and 8 (6, 10), respectively, whereas the SCL-90 total scores were 147.972 ± 0.20 and 113.151 ± 5.59. Both PSQI and SCL-90 scores were significantly higher in the PTSD group than in the non-PTSD group (all *P* < 0.001), indicating that poorer sleep quality and greater global psychological distress were strongly associated with PTSD status at the same assessment time point.

### Combined PSQI and SCL-90 assessment showed high value for early identification of PTSD in emergency trauma surgery patients

**Figure 2:**
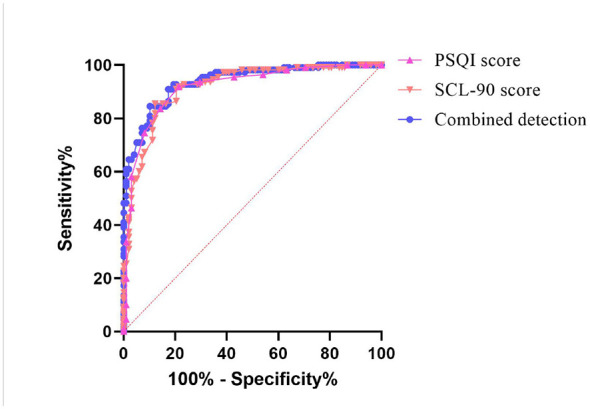
*Discriminatory value of PSQI, SCL-90, and their combined assessment for PTSD identified in patients undergoing emergency trauma surgery*. The discriminatory value of PSQI scores, SCL-90 scores, and the combined indicator for PTSD status in patients undergoing emergency trauma surgery was analyzed using ROC curves.

To further evaluate the clinical utility of PSQI and SCL-90 for early identification of PTSD after emergency trauma surgery, ROC analysis was performed. As shown in Figure 2 and Table 2, the combined PSQI and SCL-90 model yielded the largest AUC (0.937, 95%CI: 0.895–0.966), which was significantly higher than that of PSQI alone (P=0.049) and SCL-90 alone (*P* = 0.014). The optimal cut-off values were 11.5 for PSQI, 128.5 for SCL-90, and 0.462 for the combined probability score.

**Table 2:** Discriminatory value of PSQI scores, SCL-90 scores, and their combined assessment for PTSD identified in patients undergoing emergency trauma surgery.

Indicator	Cut-off	Sensitivity	Specificity	AUC	95% CI
PSQI score	11.5	78.57%	91.82%	0.917	0.871–0.951
SCL-90 score	128.5	87.76%	85.45%	0.916	0.870–0.950
Combined model	0.462	89.80%	84.55%	0.937	0.895–0.966
PSQI vs. combined model	–				
SCL-90 vs. combined model	–				

Multiple ROC AUC comparisons were performed using the DeLong test in MedCalc software. Optimal cut-off values were determined by the Youden index.

### Positive correlations of PSQI and SCL-90 scores with PCL-C score and ISS in PTSD patients

**Figure 3:**
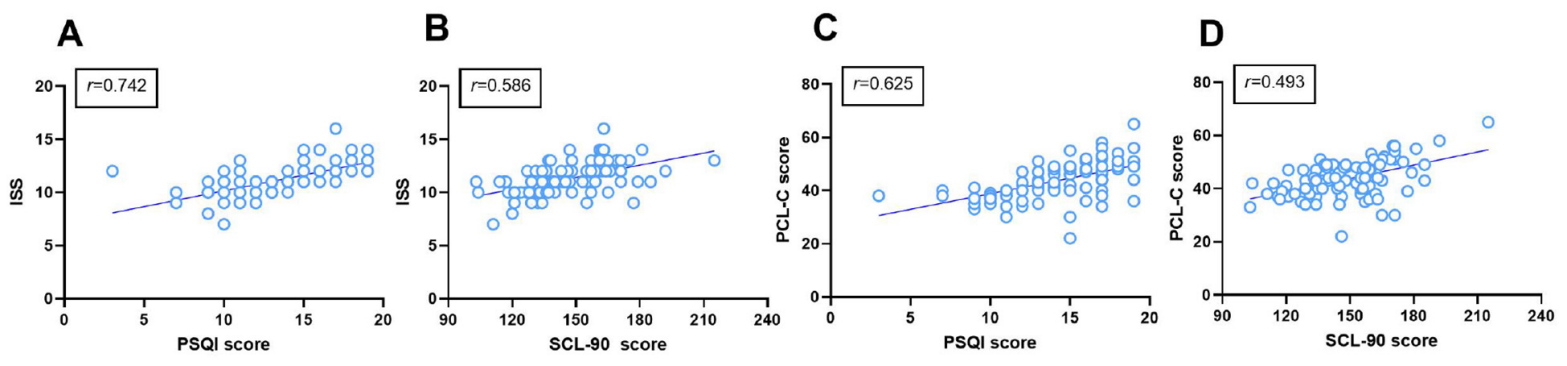
*Relationship of PSQI and SCL-90 scores with PCL-C scores and ISS in patients with PTSD*. **(A, B)** correlations of PSQI and SCL-90 scores with ISS; **(C, D)** correlations of PSQI and SCL-90 scores with PCL-C scores in PTSD patients. Pearson or Spearman analysis was used as appropriate. r denotes the correlation coefficient.

We further analyzed the correlations of PSQI and SCL-90 scores with PCL-C scores and ISS in patients with PTSD using Pearson or Spearman analysis. As shown in Figure 3, PSQI and SCL-90 scores were positively correlated with ISS (*r* = 0.742 and *r* = 0.586, respectively) and with PCL-C scores (*r* = 0.625 and *r* = 0.493, respectively; all *P* < 0.001). These findings indicate that worse sleep quality and greater overall psychological distress were associated with both greater trauma severity and more severe PTSD symptom burden.

### PSQI and SCL-90 scores were independently associated with PTSD status in patients undergoing emergency trauma surgery

Marital status, educational attainment, monthly income, ISS, PCL-C score, PSQI score, and SCL-90 score were first screened in the univariate analysis. To avoid conceptual overlap between the outcome definition and symptom severity, PCL-C was not retained in the final multivariate model, and ISS lost significance after adjustment. Multivariate logistic regression showed that educational attainment, monthly income, PSQI score, and SCL-90 score were independently associated with PTSD status (all *P* < 0.05; Table 3). These results suggest that poorer sleep quality, greater psychological distress, and socioeconomic disadvantage jointly contribute to a higher likelihood of PTSD being identified after emergency trauma surgery.

**Table 3:** Factors independently associated with PTSD status in patients undergoing emergency trauma surgery.

Variable	*B*	S.E.	Wald	*P*	OR	95% CI
Marital status	−0.044	0.303	0.021	0.885	0.957	0.529–1.733
Educational attainment	1.007	0.465	4.682	0.030	2.736	1.099–6.809
Monthly income	0.972	0.462	4.417	0.036	2.643	1.068–6.543
PSQI score	0.391	0.092	17.940	<0.001	1.479	1.234–1.773
SCL-90 score	0.063	0.017	13.794	<0.001	1.065	1.030–1.102

In the propensity score-matched sensitivity cohort, 82 matched pairs were retained. The between-group differences in PSQI and SCL-90 scores remained significant (both *P* < 0.001), the combined AUC remained high at 0.926 (95%CI: 0.876–0.961), and PSQI and SCL-90 remained independently associated with PTSD status, supporting the robustness of the main findings.

## Discussion

The present study examined patients 1 month after emergency trauma surgery and showed that PTSD status at that time point was accompanied by higher PSQI and SCL-90 scores. The combined assessment of sleep disturbance and broad psychological symptoms provided better discrimination than either scale alone. This pattern is clinically plausible because post-traumatic adaptation is multidimensional: hyperarousal, intrusive recollection, emotional dysregulation, and sleep fragmentation often co-occur after severe injury (Agorastos and Olff, 2021; Delic et al., 2021; Thakur et al., 2022). Sleep disturbance may amplify autonomic activation and impair fear extinction, whereas generalized psychological distress reflects disruption of emotional regulation, interpersonal functioning, and stress-system recovery (Agorastos and Olff, 2021; Gilbert et al., 2015; Herman et al., 2016; Serrano-Ibáñez et al., 2023). When these domains are assessed together, they may better capture the overall post-traumatic burden of emergency trauma patients and thus improve early clinical identification.

An important finding of this study is that educational attainment and monthly income were independently associated with PTSD status in addition to PSQI and SCL-90 scores. Socioeconomic factors may act as upstream determinants that influence access to health information, coping resources, treatment adherence, and the capacity to buffer stress after trauma (Agaibi and Wilson, 2005; DeCou et al., 2019; Yimer et al., 2023). Patients with lower education or lower income may also have poorer sleep, fewer social supports, and lower resilience, thereby increasing vulnerability to persistent post-traumatic symptoms. Accordingly, PTSD after emergency trauma surgery should be understood as the result of interacting psychosocial and physiological stressors rather than as an isolated reaction to injury severity alone.

Several limitations should be acknowledged. First, this was a single-center retrospective convenience sample with a modest total sample size, and the statistical power of several negative baseline comparisons was limited. Second, PSQI, SCL-90, and PTSD status were all assessed at the same postoperative time point; therefore, the present study should be interpreted as a cross-sectional comparison rather than a causal or truly predictive analysis. It is not possible to establish temporal order, and poor sleep or broad psychological distress may either precede PTSD identification or represent early manifestations of PTSD itself. Third, multiple SCL-90 dimensions, especially anxiety and depression, overlap conceptually with PTSD symptoms, which may overestimate the apparent specificity of SCL-90 for PTSD. Thus, the PSQI and SCL-90 combination should be interpreted as identifying broad post-traumatic psychological distress with high clinical relevance rather than being specific only to PTSD. Finally, although a propensity score-matched sensitivity analysis was consistent with the primary results, residual confounding and selection bias cannot be completely excluded. Prospective multicenter studies with repeated longitudinal measurements are needed to clarify temporal relationships and prognostic value.

## Data Availability

The original contributions presented in the study are included in the article/supplementary material, further inquiries can be directed to the corresponding author.
